# Communication skills in psychiatry for undergraduate students: A scoping review

**DOI:** 10.3389/fpsyt.2022.972703

**Published:** 2022-08-11

**Authors:** Filipa Novais, Licínia Ganança, Miguel Barbosa, Diogo Telles-Correia

**Affiliations:** ^1^Faculdade de Medicina, Universidade de Lisboa, Lisboa, Portugal; ^2^Centro Hospitalar Universitário Lisboa Norte, Santa Maria Hospital, Lisbon, Portugal; ^3^ISAMB – Instituto de Saúde Ambiental - Faculdade de Medicina, Universidade de Lisboa, Lisboa, Portugal; ^4^Faculdade de Psicologia, Universidade de Lisboa, Lisboa, Portugal

**Keywords:** communication skills, competencies acquiring, undergraduate, medical education - clinical skills training, psychiatry

## Abstract

Communication skills are paramount in all areas of medicine but particularly in psychiatry due to the challenges posed by mental health patients and the essential role of communication from diagnosis to treatment. Despite the prevalence of psychiatric disorders in different medical specialties, particularly in primary care settings, communication skills in psychiatry and their training are not well studied and are often not included in the undergraduate medical curriculum. Our paper explores the relevance of teaching communication competencies in psychiatry for undergraduate medical students. Our work focused on reviewing the methods for teaching communication skills to undergraduate students in Psychiatry. Eleven studies were selected to be included in this review. We found considerable heterogeneity among methods for teaching communication skills but also some common elements such as the use of simulated patients and providing feedback. This review has identified two models: the Calgary–Cambridge interview model and the Kolb cycle-based model. However, most studies still lack a theoretical background model. We believe that the inclusion of communication skills training in medical curricula is fundamental to teaching medical students general communication skills but also specific training on establishing adequate communication with psychiatric patients. However, more research is needed to determine the best method for training but also regarding its translation to patient care and cost-effectiveness.

## Introduction

The concept of communication skills (CS) has been used in the context of pre-and post-graduate training in medicine, often lacking adequate characterization. It includes specific tasks (1), skills and techniques (2), strategies (3), and some steps (4) used to establish effective communication. Most authors tend to adopt wide definitions such as “way in which a doctor can deepen the dialogue with a patient” (5), or even “direct or indirect transmission of information between two or more people that is achieved through verbal and non-verbal methods, including units of speech, visual contact, body language, gestures, and facial expressions, as well as listening methods” (6).

Communication skills have been identified as important indicators of patients‘ quality of care, regarding, for example, adherence, satisfaction with care, physical and mental health measures (2, 6), as well as indicators of doctors‘ wellbeing, including lower stress, higher quality of life, and levels of confidence) (7).

Moreover, it has been demonstrated that CS can be effectively taught, which makes them an important target in pre and postgraduate medical education (6, 8).

Communication skills can be taught in the pre- and post-graduate periods in a generic way (regarding all medical areas) or more directed to the specificities of each medical specialty (5, 6, 9). Communication skills programs have been mainly developed in specialties such as oncology, but are less frequent in psychiatry, despite being a specialty with multiple challenges from the point of view of clinical communication, namely in terms of the exploration of symptoms, communication of diagnosis, and discussion of treatment taking into account certain characteristics of psychiatric patients (9). There are specific challenges that both psychiatrists and other health professionals face when communicating with patients with mental health problems. In psychiatric settings, communicating the diagnosis of a serious mental disorder may be particularly challenging due the difficulty of explaining symptoms and etiology of disorders and the multiplicity of different treatment options and varying prognoses (10). Also, for example, disclosing the diagnosis of schizophrenia or psychotic syndromes may even more difficult due to the lack of insight and stigma associated with the disorder (10). Legal aspects, in psychotic or bipolar disorders, and particularly compulsory treatment is also a challenge for good quality communication and relationship with patients (11). Another common problem is violent or aggressive behavior, particularly in emergency settings, that may be a form of communicating distress or unmet needs. The inability to negotiate, establish clear limits in a non-confrontational style may lead to serious danger to professionals and the patient himself (12). In other settings, such as in primary care or general medical settings other frequent difficulties include dealing with patients with personality disorders. Health professionals and general medical doctors may have difficulties tolerating patients‘ affect, establishing limits in a firm but also kind way, accepting dependency and vulnerability or avoiding arguments and unreasonable requests from patients (13). Somatization disorders are among the most frequent and also amount the most difficult to manage psychiatric disorders in primary care (14). Communication difficulties may be due to the complexity of patients' needs and problems, their narratives often reflect these difficulties being fragmented and chaotic, while most doctors are trained to use simplistic and dualistic models (15).

Communication in Psychiatry involves basic skills such as empathy (16) but also more complex skills such as psychotherapeutic skills and the ability to build a therapeutic relationship (17). A paradigmatic example of the contribution of psychotherapeutic models to the development of CS training is the inclusion of transference and countertransference, concepts developed by psychodynamic authors, as fundamental CS (18, 19). More recently, other psychotherapeutic approaches, such as meditation and mindfulness, have been proposed as potential tools to improve empathy and CS in doctors (20, 21). Empathy is also another basic skill that undergraduate students and future doctors must be able to express in order to build a good quality patient-doctor relationship and effective communication (22, 23). This skill may also be trained and improved using specific CS training or specific methods (24).

Communication skills training in undergraduate teaching in the field of psychiatry allows the pluripotential physician to develop transversal communication skills, but also specific skills to support patients with psychiatric problems. In fact, up to 40% of patients in primary care suffer from a mental disorder (25). However, primary care physicians themselves assume to have difficulties in communicating with these patients (26).

In this article, we aimed to systematically review techniques and forms of implementation of CS training in psychiatry/mental health, particularly in the pre-graduate period, and, according to this evidence, suggest some orientations for the inclusion of this competence in the medical curriculum.

## Methods

The *Preferred Reporting Items for Systematic Reviews and Meta-Analyses statement (PRISMA)* guidelines were used to perform this review (27).

*Search strategies and quality of studies appraisal. Two* co-authors (F.N. and L.G.) independently searched for eligible articles through the standardized search methods for PubMed/ MEDLINE and Web of Science (Core Collection) for articles published in English or Portuguese from January 2011 until April 2022, by using Medical Subject Headings (MeSH) terms relevant in the literature regarding this topic. Due to article retrieval accessibility reasons, the online search was limited to only these two databases. Additionally, a manual bibliography search in the retrieved articles reference list was also performed. The following terms were used: *Psychiatry OR Mental Health AND communication AND Medical Students OR Medical Education OR Medical School OR Curriculum OR Clinical Clerkship OR Teaching*.

Articles were included if: (1) published in *peer review* journals; (2) described CS in Psychiatry teaching methods for medical graduate students; (3) included an objective (i.e., Objective Structured Clinical Examination OSCE) or subjective (self-report questionnaires) evaluation of CS teaching methods.

Articles were excluded if: (1) studies did not describe the curricular intervention aimed at teaching CS in psychiatry or did not include results specifically addressing these interventions; (2) studies did not report results specifically for graduate medical students in case other groups were also studied (i.e., medical doctors); (3) they were reviews, commentaries, conference communications, thesis dissertations, editorials or letters to the editor.

Two reviewers (FN and LN) independently screened titles and abstracts to potentially identify relevant papers. Then, each of the reviewers read the full text to confirm the inclusion or exclusion criteria. Disagreements were solved by consensus between the two reviewers.

### Data collection

The following descriptive data were manually extracted from eligible studies:

- Study identification.
- University name and location
- Graduate medical students' curricular year.
- CS teaching module duration.
- Training method including resources employed, types of psychiatric pathologies represented, student's tasks and types of feedback given.
- Type and results of evaluation methods.

Effect measures reported in the selected studies were not herein described as they did not pertain to this review aim.

### Study quality assessment

Quality assessment of included articles was performed using the *Medical Education Research Study Quality Instrument (MERSQI)* (28). The MERSQI is a 10-item- instrument specifically designed for medical education research. Evaluated domains include study design, sampling, type of data, evaluation instrument validity, data analysis and outcomes. Total scores vary between 5 and 18, with higher scores indicating higher study quality.

## Results

**Figure 1:**
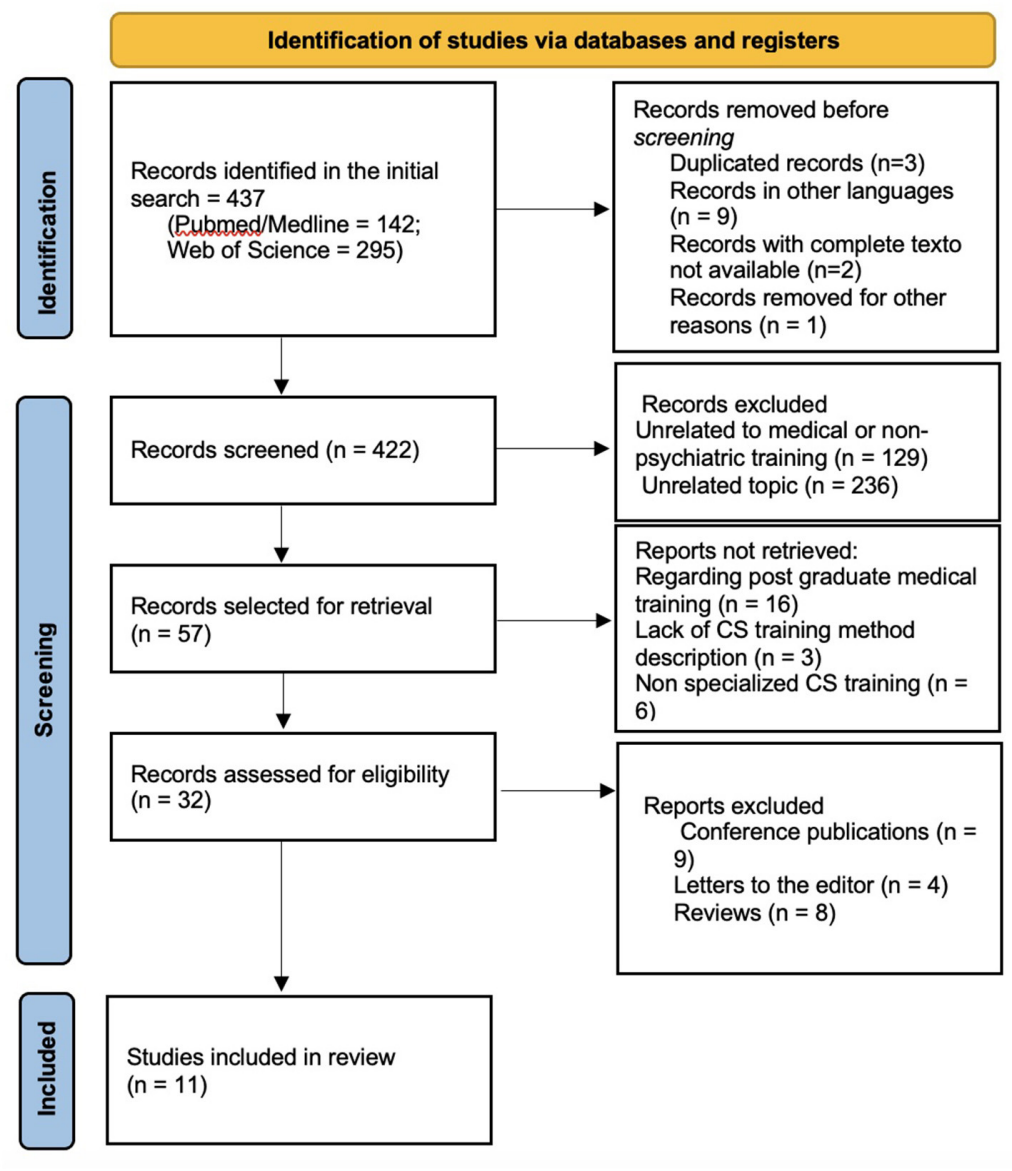
Article selection.

From a pool of 437 initially retrieved articles, 11 articles, in which CS in Psychiatry teaching methods for graduate medical students were described and evaluated, were selected for inclusion in this review. Figure 1 represents a flow diagram of the literature search and article selection algorithm according to the PRISMA guidelines.

A total of 1,920 undergraduate medical students from 11 medical schools were included. The mean MERSQI score was 11.6, ranging from 8.5 to 14. Of note, more recent studies tended to have higher MERSQI scores, indicating higher study quality. Table 1 presents a summary of the included studies according to the extracted descriptive measures. Total MERSQI scores for each study are presented in Table 2.

**Table 1:** Selected articles summary.

**References**	**Location**	**Medical training year**	**CS teaching duration**	**CS teaching method**	**Method evaluation**
Terry and Terry (14)	Iwoa University, USA	3rd/ 4th year (*n* = 118)	4 weeks, (during psychiatry rotation)	Intervention group: Didactic component + review videotaped interviews + simulated patient activity training in small groups of 3-4 students, followed by discussion and feedback Control group: Didactic component + review videotaped interviews Pathology: Suicidal ideation	Pre and post training self-report questionnaires: Significant improvement of CS in intervention group (*p* = 0.02)
Peters et al. (15)	University of Melbourne, Australia	5^th^/ 6^th^ year (*n* = 107)	6 weeks (during psychiatry rotation)	Role-play based learning method: clinical vignette, role-play (students perform both doctor and patient roles), guidelines for facilitators. Group discussion and feedback at the end. Pathologies: Anxiety, psychosis, cognitive disorders, elderly depression, eating, bipolar and personality disorders.	Self-report questionnaires: Positive global evaluations and significant improvement in CS compared to *problem-based learning* method
D'souza et al. (16)	Trinity College Dublin, Ireland	- (*n* = 129)	-	Calgary–Cambridge interview based CS online training on the SkillSims^TM^ platform. Individualized (expert) and automatic feedback.	Automatic scores positively correlated with clinical decision making and knowledge of the Calgary–Cambridge model of consultation.
Priebe and Mccabe (17)	Medical University of Vienna	4th (*n* = 529 students + 29 faculty)	-	Use of standardized patients (SPs)(actors) in role-play. 4–6 patient-student interactions per session, 20 students per group. Students and faculty reflected and gave feedback on the quality of SP's role-play. Pathologies: depression/ suicidal ideation, somatoform, anxiety and borderline personality disorders.	Self-report questionnaires: global positive evaluation of quality of SP's role play and feedback. High satisfaction of students and feedback
Hughes (18)	National Cheng Kung University in Tainan, Taiwan	2^nd^ / 5th (*n* = 208)	-	2^nd^ year – 3 components: (1) lecture on key communication components; (2) 6 problem- based learning sessions with clinical vignettes and role-play; (3) student communication with patient and family for 1h, followed by a written report. 5^th^ year – 6 weeks psychiatric rotation with lectures and training with patients. Final CS evaluation using the mini-Clinical Evaluation Exercise (mini-CEX)	Evaluation of performance of 2^nd^ year students correlated with clinical interview CS performance in the 5^th^ year
Datta-Barua and Hauser (19)	Aarhus University, Denmark	4th (*n* = 102)	4 weeks (during psychiatry rotation)	Intervention group: preparatory lecture with video case of doctor performing diagnostic interview with simulated patient Control group: conventional preparatory lecture with text-based material	27-item Self-Efficacy in Patient-Centeredness Questionnaire: showed increased scores for intervention group (*p* = 0.02)
Sundling et al. (20)	University of Bristol, United Kingdom	2^nd^, 3^rd^/ 4^th^ (*n* = 23)	Five 2h-lectures (during psychiatry rotation)	CS course. Each lecture: introduction and review of learning objectives; psychiatry residents facilitated role-play in small 3–5 students' groups; specific tasks (i.e., risk assessment); colleagues and facilitators feedback; homework and next session preparation. Students evaluated by the OSCE Pathologies: depression, suicidal ideation, anxiety, psychosis, *delirium*	Self-report questionnaire: All students considered course beneficial, no negative feedback given. Increased confidence in students' ability to assess not only psychiatric patients, but also medical and surgical ones.
Chen et al. (21)	St. George University of London, United Kingdom/ Tel-Aviv University, Israel	3rd (n=42)	1 day of training + 1 day of actual patient interview.	One day of training with simulated patients (2 clinical vignettes followed by discussion in groups of 2-7 students). Each student performed at least one role-play and observed at least 2. Sessions were recorded and given to students with a list of questions for reflection. One week after training students were evaluated by interviewing actual patients.	Pre and post training evaluation using the Four Habits Coding Scale and the Psychiatric Interview Coding Scale. A significant improvement of scores was found (*p* < 0.001 *p* = 0.002, respectively). High levels of satisfaction and confidence with training also reported.
Hashim (22)	University of Bordeaux, France	4th year (*n* = 35)	35min	Psychiatric interview with virtual simulated patients based on pre-determined scenarios with several options for the student to select leading to a single outcome. Automatic feedback was given to students regarding verbal empathy. Emotions recognition software evaluated non-verbal empathy.	Globally elevated scores. Empathy related scores significantly higher in students whom had previously observed psychiatric interviews (*p* = 0.006).
Vogel et al. (23)	Uniformed Services University, Maryland, USA	- (*n* = 321)	5 weeks (during psychiatry rotation)	Group 1: traditional model – initial discussion, interview with simulated patient, checklist completion Group 2: Kolb cycle-based model – abstract conceptualization with discussion of diagnosis and communication difficulties, active experimentation with simulated patients, concrete experience with feedback, reflexive observation)	Significantly better OSCE performance in the Kolb cycle-based model (*p* < 0.001)
Cairns et al. (24)	Friedrich-Alexander-University of Erlangen-Nuremberg, Erlangen, Germany	4th (*n* = 306)	1 week (5 days of clerkship+ 1-day OSCE)	45 min theoretical seminar; 2–3 simulated or real patient interviews; feedback from colleagues, actors, and faculty; self-evaluation quiz; Pathologies: dementia, psychosis, depression, bipolar, adjustment, obsessive-compulsive, and anxiety disorders, sexual dysfunction	Online students reported bigger development of CS on the self-report questionnaire. OSCE scores did not differ between online or on-site groups

OSCE objective structured clinical examination.

**Table 2:** Study quality scoring (Medical Education Research Study Quality Instrument-MERSQI).

**Study**	**MERSQI score**
Terry and Terry (14)	11
Peters et al. (15)	9
D'souza et al. (16)	8.5
Priebe and Mccabe (17)	10
Hughes (18)	10.5
Datta-Barua and Hauser (19)	10
Sundling et al. (20)	8.5
Chen et al. (21)	11.5
Hashim et al. (22)	11.5
Vogel et al. (23)	13
Cairns et al. (24)	14

Considering methods for CS training, 10 out of the 11 studies included simulated patients in the training of CC skills, either face-to-face or in virtual formats.

Studies comparing virtual and face-to-face models showed equivalent effectiveness.

Use of the role-play technique and feedback given by the participants, facilitators and even automatically was also common in most studies. Many of these included a theoretical component and the use of teaching materials for pre-session preparation and further study.

Two theoretical models were identified on which the structuring of some of these methods of teaching CS in Psychiatry was based: (1) the Calgary–Cambridge interview model and the Kolb cycle-based model. The former is based on the use of the Calgary-Cambridge Referenced Observation Guides, designed to outline and structure the teaching of CS in medical training and its inclusion in a comprehensive patient-centered model. These guides present a list of CS and propose a structure of the interview in the form of diagrams, in which the following steps are represented: initiating the session, gathering information, physical examination, explanation and planning, and closing the session. In each of these sections, directions for proper verbal communication are given. The authors of the model also propose techniques to be used throughout the interview to build the therapeutic relationship, such as using appropriate non-verbal language, relationship development and patient involvement (29–31); (2) The Kolb cycle concerns the continuous process that allows experiential learning through experience-simulation, being a global method of learning rather than specifically aimed at learning CS. It includes four stages that form a cycle: concrete experience, reflective observation, abstract conceptualization and active experimentation (32). Obtaining student-centered feedback is essential for closing the loop that reinforces learning and allows for active experimentation with new ideas. However, as demonstrated by the study by Meyer et al. (33) this model can be adapted to the teaching of CS. In fact, it showed better results when compared to a more traditional approach to teaching CS, only with the simulated patient interview and completion of a checklist (33–35).

Regarding the duration of CS training, it ranged from 6 weeks (part-time) to 1 day (full-time).

As for the pathologies or clinical situations represented by the simulated patients, these are not described in all articles. However, the following were included: Depression, suicidal ideation, Somatoform Disorder; Anxiety Disorder, Borderline Personality Disorder, Delirium, Dementia, Psychosis, Mania, Adaptation Disorder, Eating Behavior Disorder, Obsessive-Compulsive Disorder and Sexual Dysfunction. It is also worth mentioning that some studies used more complex situations, which seek to approach clinical practice, such as depression in elderly patients hospitalized in Orthopedics, obtaining consent in mental illness (36) patients with depression, drug use and who recently made a suicide attempt and patient with Post Traumatic Stress Disorder, conical pain and refusal to take medication (37).

In 4 of the included studies, the assessment was subjective. The rest had objective or mixed assessments, with the OSCE being the most common instrument to assess CS in undergraduate training. Overall positive results in both objective and subjective evaluations have been shown in all selected studies.

Only one study by Davies et al. (38) indicated the expenditures and resources required, specifically, for the implementation of the model. None of these studies evaluated the cost-effectiveness of these training methods.

## Discussion

Despite considerable heterogeneity regarding the methods employed for training CS in psychiatry to undergraduate medical students, a positive impact on CS students' performance satisfaction or confidence was reported by all authors.

There are several common elements that may contribute to these global positive results, in particular the use of simulated patients that has been previously reported as an effective method to train CS (39). However, most of the evidence comes from low to moderate-quality studies and it is still not clear how this method converts to improvements in patients' care (40, 41).

When comparing virtual vs. face-to-face simulated patients no difference was shown, suggesting it may be considered as a useful resource in contexts that do not allow for the traditional face-to-face format. Previous authors have also confirmed that digital education is similar to traditional education in CS training (42, 43).

Giving feedback to students is also a central component of the training. It is not clear, however, who should give this feedback. Both this review and results from previous literature seem to support that feedback may always be beneficial independently of who provides it (23, 44–46).

Most studies did not report a theoretical background despite the fact that there are well-studied models cited in literature and it could potentially structure the training and drive decisions regarding the inclusion of CS (7). In the studies included in this review, two models have been identified and could potentially be relevant for future studies. In other medical areas and undergraduate general CS training, other models, such as the COMSKIL, have been effectively developed and used (47). From this gold standard model developed in Oncology, Ditton-Phare et al. (48) developed the ComPsych directed for postgraduate CS training in Psychiatry (48). This interesting model has not been adapted yet to undergraduate training.

No comparative or review studies were found discussing what are the most important psychiatric disorders to be represented by simulated patients. Nonetheless, it seems reasonable that, for the training of the pluripotential doctor, at least, the most frequent psychiatric disorders or those that are more frequent in primary care, such as depression, anxiety, alcohol and substance misuse, somatization disorders should be included (49–51). Other pathologies, due to their specificity or particular difficulties, could also be selected for training, such as psychotic or sexual disorders (10, 52).

It is also important to discuss the applicability of the methods employed in these studies. Most of them essentially require human resources, namely the active involvement of facilitators, actors, psychiatrists, psychiatric residents and physical resources, such as computer material and meeting spaces. Countries with more limited economic resources may find it difficult to hire professional actors or purchase electronic equipment, for example. Different types of simulated patients are mentioned, namely, psychiatric interns or the students themselves who can play the role of either patient and the doctor, or both, facilitating access and availability of human resources (36, 38). As is understandable, more time-limited interventions, such as the one proposed by Amsalem et al. (37), may have advantages in terms of ease of implementation and use of resources, however, no comparative studies were found to study this issue. In fact, concerns about the feasibility, effectiveness, and cost-effectiveness of CS training have been raised by previous authors and still need to be addressed (39–41).

Some limitations concerning this review article must be considered. Firstly, the choice of a shorter time horizon may have limited the inclusion of potentially interesting studies in this area, however, this horizon also allows for a more detailed evaluation of the most recent forms of CC training. The main limitations of the included studies were identified as the fact that most included only one group, thus not allowing the comparison between different methods. Moreover, in many studies, the assessment is made by the student himself, subjectively, through self-completion questionnaires. The use of self-assessment questionnaires may limit the interpretation of the method's effectiveness, particularly regarding its repercussion in practice with real or simulated patients. Many of the studies also included small samples and students with different levels of learning and experience (different years and with or without a previous clinical internship in Psychiatry). The MERSQI score was also relatively low, particularly in two lower-quality studies. These limitations may impact the overall interpretation of the results. Lastly, regarding article retrieval methodology, even though Pubmed and Web of Science are comprehensible databases, being among the most commonly used ones, expanding the article search to other databases might have resulted in additional articles eligible for inclusion in this review.

Despite the limitations considered, this review allows the identification of CS teaching methods that could be effectively integrated into undergraduate medical training.

## Conclusions

In the most recent studies, different formats of teaching CS in Psychiatry were tested in undergraduate medical training, although the basic theoretical model is not always specified, the Kolb cycle and the Calgary-Cambridge interview models stand out, as the most frequently cited in the literature. These two methods, as well as other less structured ones, seem to consistently contribute to an improvement in the teaching of CS both objectively and subjectively. The use of simulated interviews, associated with student performance feedback and discussion by the faculty, may contribute to its effectiveness. There is no specific method that has been shown to be more effective in teaching CS in the disciplinary area of Psychiatry. The elements that can be integrated into the medical curriculum that most contribute to the learning of CS in Psychiatry still need to be developed and tested. We propose that future studies on CS training be based on adequate theoretical models and test its translation into patient care as well as its cost-effectiveness.

## Author contributions

FN and DT-C conceived and designed the manuscript. FN wrote the first draft. FN and LG were responsible for data collection. LG and MB critically reviewed the manuscript. DT-C was the responsible for the final review and quality assessment. All authors contributed to the article and approved the submitted version.

## Conflict of interest

The authors declare that the research was conducted in the absence of any commercial or financial relationships that could be construed as a potential conflict of interest.

## Publisher's note

All claims expressed in this article are solely those of the authors and do not necessarily represent those of their affiliated organizations, or those of the publisher, the editors and the reviewers. Any product that may be evaluated in this article, or claim that may be made by its manufacturer, is not guaranteed or endorsed by the publisher.

## References

[1] Makoul G. The SEGUE Framework for teaching and assessing communication skills. Patient Educ Couns. (2001) 45:23–34. 10.1016/S0738-3991(01)00136-7 11602365

[2] Roter DL Hall JA. Doctors Talking With Patients/Patients Talking With Doctors: Improving Communication in Medical Visits. Westport, CT, US: Auburn House/Greenwood Publishing Group (1992). xii, p. 203.

[3] Razavi D Delvaux N Marchal S Durieux JF Farvacques C Dubus L. Does training increase the use of more emotionally laden words by nurses when talking with cancer patients? A randomised study. Br J Cancer. (2002) 87:1–7. 10.1038/sj.bjc.6600412 12085247 PMC2364281

[4] Baile WF Kudelka AP Beale EA Glober GA Myers EG Greisinger AJ. Communication skills training in oncology. Description and preliminary outcomes of workshops on breaking bad news and managing patient reactions to illness. Cancer. (1999) 86:887–97. 10463990

[5] Bylund CL Brown R Gueguen JA Diamond C Bianculli J Kissane DW. The implementation and assessment of a comprehensive communication skills training curriculum for oncologists. Psychooncology. (2010) 19:583–93. 10.1002/pon.1585 19484714

[6] Ditton-Phare P Loughland C Duvivier R Kelly B. Communication skills in the training of psychiatrists: A systematic review of current approaches. Aust N Z J Psychiatry. (2017) 51:675–92. 10.1177/0004867417707820 28462636

[7] Cegala DJ Lenzmeier Broz S. Physician communication skills training: a review of theoretical backgrounds, objectives and skills. Med Educ. (2002) 36:1004–16. 10.1046/j.1365-2923.2002.01331.x 12406260

[8] Brown RF Bylund CL. Communication skills training: describing a new conceptual model. Acad Med. (2008) 83:37–44. 10.1097/ACM.0b013e31815c631e 18162748

[9] Schneider B Scissons H Arney L Benson G Derry J Lucas K. Communication between people with schizophrenia and their medical professionals: a participatory research project. Qual Health Res. (2004) 14:562–77. 10.1177/1049732303262423 15068580

[10] Farooq S Johal RK Ziff C Naeem F. Different communication strategies for disclosing a diagnosis of schizophrenia and related disorders. Cochrane Database Syst Rev. (2017) 10:CD011707. 10.1002/14651858.CD011707.pub2 29064090 PMC6485682

[11] Pestana-Santos A Loureiro L Santos V Carvalho I. Patients with schizophrenia assessing psychiatrists' communication skills. Psychiatry Res. (2018) 269:13–20. 10.1016/j.psychres.2018.08.040 30145294

[12] Harwood RH. How to deal with violent and aggressive patients in acute medical settings. J R Coll Physicians Edinb. (2017) 47:94–101. 10.4997/JRCPE.2017.218 28675195

[13] Miller MC. Personality disorders. Med Clin North Am. (2001) 85:819–37. 10.1016/S0025-7125(05)70342-4 11349486

[14] Terry DL Terry CP. Addressing mental health concerns in primary care: practices among medical residents in a rural setting. J Clin Psychol Med Settings. (2019) 26:395–401. 10.1007/s10880-019-09609-3 30758698

[15] Peters S Rogers A Salmon P Gask L Dowrick C Towey M. What Do Patients Choose to Tell Their Doctors? Qualitative analysis of potential barriers to reattributing medically unexplained symptoms. J Gen Intern Med. (2008) 24:443. 10.1007/s11606-008-0872-x 19089505 PMC2659146

[16] Rasquinha PC D'souza TL Jain A Kulkarni V Pai K. Effect of a single-session communication skills training on empathy in medical students. Acad Psychiatry. (2020) 44:289–94. 10.1007/s40596-019-01158-z 31811627

[17] Priebe S Mccabe R. Therapeutic relationships in psychiatry: the basis of therapy or therapy in itself? Int Rev Psychiatry. (2008) 20:521–6. 10.1080/09540260802565257 19085408

[18] Hughes P. Transference and countertransference in communication between doctor and patient. Adv Psychiatr Treat. (2000) 6:57–64. 10.1192/apt.6.1.57

[19] Datta-Barua I Hauser J. Four communication skills from psychiatry useful in palliative care and how to teach them. AMA J Ethics. (2018) 20:E717–723. 10.1001/amajethics.2018.717 30118421

[20] Sundling V Sundler AJ Holmström IK Kristensen DV Eide H. Mindfulness predicts student nurses' communication self-efficacy: a cross-national comparative study. Patient Educ Couns. (2017) 100:1558–63. 10.1016/j.pec.2017.03.016 28342674

[21] Chen H Liu C Cao X Hong B Huang DH Liu CY. Effects of Loving-Kindness Meditation on Doctors' Mindfulness, Empathy, and Communication Skills. Int J Environ Res Public Health. (2021) 18:4033. 10.3390/ijerph18084033 33921271 PMC8069630

[22] Hashim MJ. Patient-centered communication: basic skills. Am Fam Physician. (2017) 95:29–34. 28075109

[23] Vogel D Meyer M Harendza S. Verbal and non-verbal communication skills including empathy during history taking of undergraduate medical students. BMC Med Educ. (2018) 18:157. 10.1186/s12909-018-1260-9 29970069 PMC6029273

[24] Cairns P Pinker I Ward A Watson E Laidlaw A. Empathy maps in communication skills training. Clin Teach. (2021) 18:142–6. 10.1111/tct.13270 33034104

[25] Spitzer RL Williams JBW Kroenke K Linzer M deGruy FV III Hahn SR. Utility of a New Procedure for Diagnosing Mental Disorders in Primary Care: The PRIME-MD 1000 Study. JAMA. (1994) 272:1749–56. 10.1001/jama.1994.03520220043029 7966923

[26] Stensrud TL Mjaaland TA Finset A. Communication and mental health in general practice: physicians' self-perceived learning needs and self-efficacy. Ment Health Fam Med. (2012) 9:201–9. 23997826 PMC3622912

[27] Page MJ McKenzie JE Bossuyt PM Boutron I Hoffmann TC Mulrow CD. The PRISMA 2020 statement: an updated guideline for reporting systematic reviews. BMJ. (2021) 372:n71. 10.1136/bmj.n71 33782057 PMC8005924

[28] Reed DA Cook DA Beckman TJ Levine RB Kern DE Wright SM. Association between funding and quality of published medical education research. JAMA. (2007) 298:1002–9. 10.1001/jama.298.9.1002 17785645

[29] Kurtz SM Silverman JD. The Calgary—Cambridge Referenced Observation Guides: an aid to defining the curriculum and organizing the teaching in communication training programmes. Med Educ. (1996) 30:83–9. 10.1111/j.1365-2923.1996.tb00724.x 8736242

[30] Sommer J Lanier C Perron NJ Nendaz M Clavet D Audétat MC. teaching skills assessment tool inspired by the Calgary–Cambridge model and the patient-centered approach. Patient Educ Couns. (2016) 99:600–9. 10.1016/j.pec.2015.11.024 26680755

[31] Kurtz S Silverman J Benson J Draper J. Marrying content and process in clinical method teaching: enhancing the Calgary–Cambridge Guides. Acad Med. (2003) 78:802–9. 10.1097/00001888-200308000-00011 12915371

[32] Kolb DA Wolfe DM. Professional Education and Career Development: A Cross Sectional Study of Adaptive Competencies in Experiential Learning. Lifelong Learning and Adult Development Project. Final Report. (1981). Available online at: https://eric.ed.gov/?id=ED209493 (accessed February 1, 2022).

[33] Meyer EG Battista A Sommerfeldt JM West JC Hamaoka D Cozza KL. Experiential learning cycles as an effective means for teaching psychiatric clinical skills via repeated simulation in the psychiatry clerkship. Acad Psychiatry. (2021) 45:150–8. 10.1007/s40596-020-01340-8 33169304 PMC7652584

[34] Abulebda K Auerbach M Limaiem F. Debriefing Techniques Utilized in Medical Simulation. StatPearls. Treasure Island (FL): StatPearls Publishing (2022). Available online at: http://www.ncbi.nlm.nih.gov/books/NBK546660/ (accessed February 4, 2022). 31536266

[35] Khajuria A Mathew Jr J. Simulation Training and Skill Assessment in Orthopedic Surgery. StatPearls. Treasure Island (FL): StatPearls Publishing (2022). Available online at: http://www.ncbi.nlm.nih.gov/books/NBK559080/ (accessed February 4, 2022). 32644506

[36] King J Hill K Gleason A. All the world's a stage: evaluating psychiatry role-play based learning for medical students. Australas Psychiatry. (2015) 23:76–9. 10.1177/1039856214563846 25512966

[37] Amsalem D Gothelf D Soul O Dorman A Ziv A Gross R. Single-Day Simulation-Based Training Improves Communication and Psychiatric Skills of Medical Students. Front Psychiatry. (2020) 11:221. 10.3389/fpsyt.2020.00221 32265762 PMC7099001

[38] Davies J Churchhouse G Buckley M. Improving the provision of clinical skills teaching for undergraduate medical students during their psychiatry placement: a trainee-led quality improvement project. Australas Psychiatry. (2020) 28:101–5. 10.1177/1039856219871874 31535561

[39] Kerr D Ostaszkiewicz J Dunning T Martin P. The effectiveness of training interventions on nurses' communication skills: a systematic review. Nurse Educ Today. (2020) 89:104405. 10.1016/j.nedt.2020.104405 32244125

[40] Kaplonyi J Bowles KA Nestel D Kiegaldie D Maloney S Haines T. Understanding the impact of simulated patients on health care learners' communication skills: a systematic review. Med Educ. (2017) 51:1209–19. 10.1111/medu.13387 28833360

[41] Papageorgiou A Loke YK Fromage M. Communication skills training for mental health professionals working with people with severe mental illness. Cochrane Database Syst Rev. (2017) 6:CD010006. 10.1002/14651858.CD010006.pub2 28613384 PMC6481374

[42] Kyaw BM Posadzki P Paddock S Car J Campbell J Tudor Car L. Effectiveness of digital education on communication skills among medical students: systematic review and meta-analysis by the digital health education collaboration. J Med Internet Res. (2019) 21:e12967. 10.2196/12967 31456579 PMC6764329

[43] Shorey S Ang E Yap J Ng ED Lau ST Chui CK. Virtual counseling application using artificial intelligence for communication skills training in nursing education: development study. J Med Internet Res. (2019) 21:e14658. 10.2196/14658 31663857 PMC6913997

[44] Qureshi AA Zehra T. Simulated patient's feedback to improve communication skills of clerkship students. BMC Med Educ. (2020) 20:15. 10.1186/s12909-019-1914-2 31941466 PMC6964074

[45] Faucett EA McCrary HC Barry JY Saleh AA Erman AB Ishman SL. High-quality feedback regarding professionalism and communication skills in otolaryngology resident education. Otolaryngol Head Neck Surg. (2018) 158:36–42. 10.1177/0194599817737758 29065274

[46] Mazor KM King AM Hoppe RB D'Addario A Musselman TG Tallia AF. Using crowdsourced analog patients to provide feedback on physician communication skills. Patient Educ Couns. (2021) 104:2297–303. 10.1016/j.pec.2021.02.047 33715944

[47] Gebhardt C Mehnert-Theuerkauf A Hartung T Zimmermann A Glaesmer H Götze H. COMSKIL: a communication skills training program for medical students. GMS J Med Educ. (2021) 38:Doc83. 10.3205/zma001479 34056072 PMC8136352

[48] Ditton-Phare P Sandhu H Kelly B Loughland C. ComPsych communication skills training: Applicability of simulated patients in psychiatry communication skills training. Australas Psychiatry. (2022). 10.1177/10398562211067199. [Epub ahead of print]. 35138955

[49] Reho TTM Atkins SA Talola N Viljamaa M Sumanen MPT Uitti J. Frequent attenders in occupational health primary care: a cross-sectional study. Scand J Public Health. (2019) 47:28–36. 10.1177/1403494818777436 29806549

[50] Palzes VA Parthasarathy S Chi FW Kline-Simon AH Lu Y Weisner C. Associations between psychiatric disorders and alcohol consumption levels in an adult primary care population. Alcohol Clin Exp Res. (2020) 44:2536–44. 10.1111/acer.14477 33151592 PMC7756330

[51] Leutgeb R Berger S Szecsenyi J Laux G. Patients with somatoform disorders: More frequent attendance and higher utilization in primary Out-of-Hours care? PLoS ONE. (2018) 13:e0202546. 10.1371/journal.pone.0202546 30161150 PMC6116940

[52] Kingsberg SA Knudson G. Female sexual disorders: assessment, diagnosis, and treatment. CNS Spectr. (2011) 16:49–62. 10.1017/S109285291200017X 24725342

